# Barriers and Facilitators to Adoption of Genomic Services for Colorectal Care within the Veterans Health Administration

**DOI:** 10.3390/jpm6020016

**Published:** 2016-04-28

**Authors:** Nina R. Sperber, Sara M. Andrews, Corrine I. Voils, Gregory L. Green, Dawn Provenzale, Sara Knight

**Affiliations:** 1 Center for Health Services Research in Primary Care, Health Services Research & Development Service, 411 West Chapel Hill Street, Suite 600, Durham, NC 27701, USA; Sara.Andrews@va.gov (S.M.A.); corrine.voils@dm.duke.edu (C.I.V.); 2 Department of Medicine, Division of General Internal Medicine, Duke University School of Medicine, 411 West Chapel Hill Street, Suite 500, Durham, NC 27701, USA; 3 VAMC, 4150 Clement Street, San Francisco, CA 94121, USA; gregory.green@va.gov; 4 Department of Veterans Affairs, Cooperative Studies Program Epidemiology Center, 411 West Chapel Hill Street, Suite 600, Durham, NC 27701, USA; dawn.provenzale@va.gov; 5 Health Services Research & Development Service, 700 S. 19th Street, Birmingham, AL 35233, USA; sara.knight@va.gov; 6 Department of Medicine, Division of Preventive Medicine, University of Alabama at Birmingham, MT 638, 1720 2nd Avenue South, Birmingham, AL 35294, USA

**Keywords:** implementation research, Lynch syndrome, qualitative methods

## Abstract

We examined facilitators and barriers to adoption of genomic services for colorectal care, one of the first genomic medicine applications, within the Veterans Health Administration to shed light on areas for practice change. We conducted semi-structured interviews with 58 clinicians to understand use of the following genomic services for colorectal care: family health history documentation, molecular and genetic testing, and genetic counseling. Data collection and analysis were informed by two conceptual frameworks, the Greenhalgh Diffusion of Innovation and Andersen Behavioral Model, to allow for concurrent examination of both access and innovation factors. Specialists were more likely than primary care clinicians to obtain family history to investigate hereditary colorectal cancer (CRC), but with limited detail; clinicians suggested templates to facilitate retrieval and documentation of family history according to guidelines. Clinicians identified advantage of molecular tumor analysis prior to genetic testing, but tumor testing was infrequently used due to perceived low disease burden. Support from genetic counselors was regarded as facilitative for considering hereditary basis of CRC diagnosis, but there was variability in awareness of and access to this expertise. Our data suggest the need for tools and policies to establish and disseminate well-defined processes for accessing services and adhering to guidelines.

## 1. Introduction

Use of genomic health services to identify Lynch syndrome (LS), or hereditary nonpolyposis colon cancer (HNPCC), the most common hereditary colon cancer, is one of the first evidence-based genomic medicine applications. Longstanding guidelines recommend that all persons younger than age 50 diagnosed with colorectal cancer (CRC) receive one or more of the following genomic services to identify LS: family history assessment, genetic counseling, molecular tumor analysis, and genetic testing [1,2,3]. Recently, the Centers for Disease Control and Prevention Evaluation of Genomic Applications for Practice and Prevention workgroup has recommended testing tumor tissue on all newly-diagnosed CRC, specifically molecular analysis for microsatellite instability by PCR (MSI) or immunohistochemistry (IHC), and, if indicated, more costly but definitive genetic testing of germline mismatch repair gene mutation [4]; this recommendation is supported by the US Multi-Society Task Force on Colorectal Cancer and the European Society of Medical Oncology [5,6]. 

Diagnosis of LS has important implications for CRC treatment and surveillance. It is suggested that patients with a LS diagnoses are offered subtotal colectomy to prevent future colorectal cancers or surveillance with colonoscopy every 1 to 2 years, in contrast to the usual surveillance every 5 to 10 years after CRC diagnosis, and that female patients are offered hysterectomy and bilateral salpingo-oophorectomy to prevent endometrial and ovarian cancers. Additionally, LS screening can have a cascading public health benefit in that at-risk relatives of cancer patients could be diagnosed [4,7]. 

Increasingly, US health care systems are implementing routine testing of CRC patients for LS, shedding light on facilitators and barriers in various settings. Schneider *et al.* interviewed frontline clinical staff and administrators within Kaiser Permanente Northwest, an integrated health maintenance organization, on the cusp of implementing a LS screening program and found that though these stakeholders sensed “a national movement” toward routine LS screening and were motivated to follow suit, they were hesitant, not knowing what other systems had actually implemented and how to change their practice to meet current guidelines [8]. A multidisciplinary team at a colorectal cancer specialty clinic designed a protocol for LS screening that included dedicated genetic counselors and a referral criteria tool; this process was successful in increasing screening adherence, though not every patient had health insurance to cover testing, minimizing overall impact [9]. In one university-based safety net health care setting, a dedicated genetics team also proved key to ensuring that IHC tests were ordered and appropriately followed on all newly diagnosed CRC cases [10]. 

The Veterans Health Administration (VHA), the largest integrated health care system in the US, has begun to develop infrastructure to incorporate genomics into healthcare for veterans, for example with establishment of a Genomic Medicine Service (GMS) in 2010. Prior evaluation of general genomic services in the VHA has revealed that expert support and clear protocols are needed to improve uptake. Scheuner *et al.* found that provider education on genetic topics and presence of an on-site genetics champion—that is, a provider with a specific interest in and commitment to incorporating genomic services in practice—have been associated with greater use of genetic consultative services [11]. Hamilton *et al.* conducted a study to identify features of genomic services (e.g., relative advantage in value, perceived complexity of service, benefits observable to users) that are important for their adoption in the VHA and illuminated a need for targeted implementation strategies to facilitate adoption beyond relying on features of the services themselves [12]. 

While previous studies illustrate several health provider and innovational factors that may influence adoption, a gap remains in understanding how provider, healthcare system, and innovation factors comprehensively may influence the multiple services that would be needed to identify a hereditary condition. This comprehensive understanding is necessary to provide a foundation for a sound implementation approach for the incorporation of genomic services in cancer care. The purpose of our study was to address this gap by identifying comprehensive factors impacting adoption of genomic services specifically for Veterans diagnosed with CRC within the VHA, focusing on four genomic services typically used together to identify risk for LS: family health history documentation, genetic counseling, molecular testing, and genetic testing. We used qualitative methods to elicit detailed, descriptive data from clinicians about their experiences with using genomic services to identify LS. Because genomic services for LS are nascent in the VHA, we used a framework based on both access to care and diffusion of innovation to guide data collection and analysis [13,14]. By applying a multi-level framework, we expanded upon research about uptake of genomic services with the VHA, considering not only how features of specific genomic services may impact uptake, but also how factors associated with individual providers and with the VHA as a healthcare system may affect adoption of these services. This study adds to the literature base on health systems’ use of genomic services in routine CRC care by offering theoretical and evidence-based strategies to facilitate adherence to national LS guidelines at multiple levels in a large, complex health system. 

## 2. Methods

We designed our data collection and analysis with two frameworks that have been used to explain factors impacting utilization of health services and diffusion of innovation, the Andersen Behavioral Model of Health Services and the Diffusion of Innovation Model [13,14,15]. The Andersen Behavioral Model of Health Services posits that structural and individual factors are key considerations in access to care and utilization of services. Structural factors include availability of genomic services and organizational characteristics that support their use (for example, values, norms, goals, ways of working). Individual factors include knowledge and attitudes as well as resources that are available to support individuals. The Diffusion of Innovation Model asserts that features of a new practice, in addition to organizational structural factors, can impact extent of its adoption: for example, innovations, in this case use of genomic services in routine clinical care, that are regarded as having clear advantage in effectiveness will be more readily adopted. By drawing from these these two frameworks we were able to elicit factors important for adoption of genomic services in the VHA and organize findings according to structural, individual and innovational domains to identify opportunities for practice change from multiple leverage points.

Semi-structured telephone interviews were conducted between 10 July 2010 to 24 September 2014 by three health services researchers, trained in social and behavioral sciences, with clinicians from different specialties who are involved with CRC patients, including oncology, gastroenterology, primary care, and pathology. Because we expected that the availability of oncology services, including associated clinical geneticist and genetic counseling services, would influence reports of barriers and facilitators potentially influencing access and diffusion of genomic services for LS, we used stratified sampling based on oncology volume to enhance the contrast between facilities that may vary widely in types of supports available for Veterans diagnosed with cancer. We included 15 VHA facilities with the highest oncology volume and 15 facilities with the lowest oncology volume determined by VHA administrative data on facility oncology volume in 2013. We excluded facilities where key study personnel were located (Durham and San Francisco). Based on the reasoning that a minimal level of oncology volume is necessary for facilities to have experience with CRC and related genomic services, we excluded facilities that had very low oncology volume (0 to 29 unique cases per year). 

We recruited key informants, service chiefs and ancillary staff identified as those most knowledgeable about genomic services for CRC at their facilities. We contacted the Chief of Staff or the Associate Chief of Staff for Research at each facility by e-mail to introduce the study and request assistance with recruitment of key informants. Chiefs or Associate Chiefs were asked to identify service chiefs and ancillary staff at their facility most knowledgeable about genomic services for CRC from specialties such as medical oncology, surgery, pathology, gastroenterology, and primary care. Each key informant was asked to identify additional participants. We planned to recruit until thematic saturation, *i.e.*, redundancy of interview information sufficient to answer research questions, and relatively equal representation across high and low oncology volume facilities and specialties.

Our interview guide was informed by our conceptual frameworks, the Greenhalgh Diffusion of Innovation Model and the Andersen Behavioral Model, and previous work on utilization of genomic services outside the VHA [16]. The final version also incorporated feedback obtained through meetings with oncologists, primary care professionals, pathologists, tumor registry staff, and genetic counselors located at the San Francisco VA Medical Center, the University of California at San Francisco, and Stanford University. Interviewers asked respondents what they knew about availability of genomic services at their VHA facility or at non-VHA facilities to identify hereditary CRC and other structural factors, such as processes to collect and use genomic information. Interviewers also asked respondents questions related to individual factors impacting use of genomic services, for example their knowledge about guidelines, and to reflect on features of services, for example by describing what resources are available to help with using genomic services. Interviews lasted 20–30 min and were audio-recorded and transcribed. Interview questions are presented in Table 1.

To analyze data, we used a directed approach to content analysis in that codes, or descriptive labels, were *a priori* based on theoretical constructs from these two frameworks and focus of interview questions [17]. The theoretical constructs that we used as codes are presented in Table 2: we adapted definitions of these constructs to our focus on genomic services. Additional codes captured interview questions, for example “obtaining results” to reflect responses to the question, “How are results reported back to the VHA facility?”, and the type of genomic service discussed, for example “family health history documentation” or “genetic counseling.” 

We aggregated coded data from each clinician interview and organized the data in a matrix with rows representing individual respondents and columns indicating theoretical domain (structural, individual, or innovational) For example, data coded as “innovation-system fit” were summarized in the structural domain column. As part of the analytic process, we assessed whether the coded data manifested as facilitators or barriers to using genomic services of family health history, clinical testing and genetic counseling in the VHA. 

We used a team approach to coding and analyzing data. Two analysts (NS, SA) independently coded transcripts and then met to compare coded text and resolve differences; a third team member (SK) participated in early coding meetings to help define application of codes. Analysts additionally worked together to further interpret data, discussing whether factors were facilitators or barriers and implications for practice change to arrive at consensus. Qualitative software, ATLAS.ti v6.2 (Berlin, Germany), was used to manage data. 

## 3. Results 

### 3.1. Overview

We interviewed 58 clinicians from five specialties: primary care (*n* = 11); oncology (*n* = 13); surgery (*n* = 7); gastroenterology (*n* = 18); pathology (*n* = 9); these clinicians were from 14 high oncology-volume and 9 low oncology-volume facilities. Themes represent clinicians’ reports of factors across low- and high-volume sites that may impact incorporation of genomic services into routine clinical care, and individual quotes are included to illustrate findings. An overview of findings associated with these factors, along with our assessment of implications from these results, is presented in Table 3. 

### 3.2. Key Themes for Genomic Services by Theoretical Domain

#### 3.2.1. Family Health History Documentation: Routinely Collected, but without Guideline-Informed Policy or Template and Variable by Specialty

*Structural*: Clinicians across specialties reported routine collection of family health information, but adoption was hampered by lack of policy to standardize collection according to recommended clinical guidelines. As a result, clinicians said that they did not have clear, specific criteria for inquiring about and acting upon family history. They did not typically probe patients about 2nd or 3rd degree relatives or age of relatives at diagnosis and instead relied on patients to offer the extent of their family health history. 

*Individual*: Content of family history collected was dependent on individual visit and clinician focus. For example, primary care clinicians were likely to ask patients at the initial visit about their general family health history, focusing on more familiar conditions, such as diabetes. Gastroenterologists and oncologists were likely to ask about family history of CRC, uterine, and gastric cancer, but with limited detail. Gastroenterologists specifically spoke of increased volume from open access screening colonoscopy, in which patients are referred directly to colonoscopy without previous clinical consultation, hindering detailed family history assessments. As one gastroenterologist described,

“Frankly, we sometimes are doing 25 endoscopies a day… I ask, “Any family history of bowel problems?” If they say “Yes,” often that’s why they are here for early screening that the primary care doctor has figured out. If they say “No,” I don’t delve any deeper. We’ve got to move on.”

*Innovational*: Clinicians asserted that a tool would help guide them in obtaining family health history according to latest standards. As one primary care clinician said,

“I know that…some providers have their own that they’ve developed, but I don’t know of a standardized tool…I think what it does, it lends to less complete information, because they are not as apt to ask all the questions if there is not a standardized tool. We know that for a fact in almost every area.”

#### 3.2.2. Clinical Testing: Molecular Tumor Testing Regarded as Available and Advantageous Prior to Genetic Germline Testing, Though Used Seldom 

*Structural*: A frequently cited barrier to testing for LS within VHA was that clinicians see few cases that raise suspicion of familial CRC, because the patient population tends to be older. Additionally, procedures for accessing molecular and genetic testing were not clear in every case, in part because services are not clinically indicated often enough within the patient population to develop request and approval routines. As one gastroenterologist said,

“We don’t have a system that’s really down pat, and I’ve got to be honest with you, it’s very laborious to try to find the right person to ask, and, then, to get them to agree… to send this out. So every time we have to do it, we have to stumble through it again… we never know exactly what process is the right process to take. Every time, it’s like reinventing the wheel.”

*Individual:* Although respondents were able to articulate value of Lynch Syndrome testing, it was acknowledged that having clinicians on board with a particular interest in this approach was important for widespread, routine use. For example, respondents at two sites said that their facility had instituted testing of all new cancer cases when clinicians had advocated for the practice. In many sites, though, clinicians just were not aware of current LS guidelines, and awareness seemed to vary by specialty or clinic; as with family health history, many primary care clinicians were much less familiar with guidelines, indicating that they knew vaguely about familial colon cancer but not specific clinical criteria, and several indicated interest in learning more. One primary care clinician said,

“You know, I think some things we’re better at thinking about genetics than others. And I don’t know if anybody is particularly good about thinking about genetic testing for colorectal screening really, or for cancer risk... I don’t know if it’s been just not that well publicized, or, you know, like breast cancer gets all the PR.”

Clinicians said that awareness by pathologists about need and process for molecular testing was critical because they are instrumental in the use of these services. One pathologist said,

“If they (pathologists) don’t contribute to it, or buy into it, or commit to it, then those tests will never be sent. Because it’s not really in anyone’s radar to get it done. It’s not something that’s second nature to clinicians yet.”

*Innovational:* Clinicians identified relative advantage to using molecular testing as part of routine CRC care, specifically utility for making decisions about CRC care and accuracy in assessing a possible hereditary component, compared to relying on family history alone. One pathologist described advantage of using molecular testing as *“…being able to detect patients who might be familial colon cancer patients, where family history was either not available or not apparent.”* Another justification for using molecular testing for LS was relatively low cost and definitive guidelines, compared to other conditions (e.g., neurological diseases) for which testing is expensive and less guideline-directed.

#### 3.2.3. Genetic Counseling: Desire for Expert Support Such as Genetic Counseling to Help with Adherence to Guidelines and Interpretation of Results, but Variability in Access to and Awareness of Expertise 

*Structural:* Clinicians who considered genetic testing for CRC care regarded GMS, which provides telegenetic counseling, as facilitative by improving access to genetics experts; those who used non-VHA counselors noted problems with notification about approvals and results, though in one case a provider described having a physician assistant available to monitor “non-VA consulted procedures…to make sure the loop is closed…” Some said that GMS improved access to counseling with a standard inter-facility consult for referrals instead of a case-by-case formal approval process, which can delay services. Also, clinicians said that telecounseling would be useful for patients who refuse to go outside of the VHA or to travel to another VHA facility for these services. Despite structural advantages to having telecounseling available within the VHA, some clinicians who were aware of this service were unable to use it as a resource; at the time that interviews were conducted, only 2 facilities in our sample had an agreement in place for the VHA GMS to provide telecounseling. 

*Individual*: Many clinicians said that they lacked expertise in genomics altogether and had little experience with LS; while they were capable of providing limited counseling to patients, for example to initiate testing, they wanted assistance with interpreting results. As one gastroenterologist said,

“If somebody has familial polyposis, I certainly will inform them about what the possibilities are of them, or a sibling or a child having the disease, and what the implications of the disease are for them and their family, and the possibility of getting colon cancer and so forth. I mean, in general strokes I will tell them that so they’re informed about that kind of stuff. But I won’t do any formal genetic counseling, you know, to give them exact probabilities, because I’m just not an expert at that.”

*Innovational:* Key informants appreciated having support to counsel patients about tests for LS and interpretation of test results, though the source of this support varied across facilities. Clinicians at four facilities reported having locally designated navigators to help with tracking referrals and testing for cancer care, including at one location a program to counsel cancer patients over the telephone, resulting in attention to screening for LS. Others relied on experts within the VHA but outside of their facility, for example at other VHA facilities or GMS, or, when counselors were not available from within the VHA, from academic affiliates. 

## 4. Discussion

Our findings revealed that VHA clinicians who lacked information about genomics of CRC, particularly in primary care, were less likely to apply guidelines to assess risk for this condition. Clinicians who were familiar with hereditary CRC generally regarded tumor testing and expert genetic support, *i.e.*, geneticists and genetic counselors, as advantageous for clinical care of CRC patients; however, they did not necessarily perceive a need for this infrastructure given their older age patient population. These findings underscore a need for education about LS testing as well as innovative tools and incentives to facilitate systematic guideline-informed screening and coordination between specialties. 

Though family history information can be informative in assessing risk for LS and other genetic diseases, there are numerous barriers to effectively employing in practice, including limited time for detailed questioning, inaccuracy of patient-reported information, and clinical priorities [18]. Indeed, we found inconsistent patterns of family history elicitation, despite clear guidelines, due in part to lack of standardized questions. Tools, such as patient-facing Web based programs, are needed to facilitate thorough and systematic collection of family health history by non-genetic specialists [19]. 

One challenge to implementing genomic services for colorectal cancer within the VHA is differences between facilities in priorities and resources; this was reflected in our finding that some sites reported testing all new colorectal cancers whereas others did not. Those who did not employ routine testing cited low incidence among the older VHA population, rendering it low priority; however, without routine testing, true LS incidence among VHA users may not be known. Clinicians at sites with a higher level of attention to LS reported having a designated role to identify cancer risk and manage cancer cases and, in some cases, a clinical champion. Cohen *et al.* described a process for implementing systematic tumor screening at a colorectal cancer specialty clinic, in which a clinic nurse would track tumor test results and notify all providers involved in a patients’ CRC care, not just the ordering provider; results were reviewed by genetic counselors via a shared e-mail inbox, with abnormal results triggering a genetic counseling referral, reducing provider interpretation variability and increasing genetic counseling referrals [9]. Dedicated processes such as this to facilitate coordination and collaboration are important to ensure that clinician requests are tracked and cases do not fall through the cracks [11].

Our findings indicate that a centralized telegenetic counseling service could facilitate systematic implementation of a VHA LS program by improving access to genetic counselors from local facilities. At the time of this study, GMS had established linkages with about one-third of the 23 Veterans Integrated Service Networks (VISNs). There is an interest in this type of service from VHA clinicians, who said that benefits included not only availability of genomics experts but also a user-friendly inter-facility consult process. Marquez *et al.* found a high rate of appropriate follow-up among primarily underserved patients in a university based safety-net health system and attributed this success to a delivery model in which a genetics team met patients at their regular clinic, with familiar staff and facilities [10]. Though an “emerging model” of having genetic specialists embedded within a clinic, such as described in the Marquez study, differs from a “traditional” model of providing genetic counseling from a center [11] , for example GMS within VHA, both models enable patients to receive genetic counseling from their regular facility rather than having to visit a separate entity. 

We selected facilities with high and low oncology volume to maximize our ability to see a range of barriers and facilitators. While we had wanted originally to stratify by utilization of genomic services, we found that utilization was low across facilities and it was not possible to contrast barriers and facilitators according to utilization. Our sampling plan resulted in a reasonable range of barriers and facilitators, yet we found few discernable differences according to oncology volume. This apparent lack of difference could be related to respondent reports at both high and low oncology volume sites that they did not frequently encounter patients with LS, thus rendering it lower priority regardless of oncology volume; instead greater attention to LS at a local level seemed to depend on individual clinician interest or local clinical champion. It may be valuable to examine barriers and facilitators in future studies if it is possible to identify settings that vary in volume of genomic services utilization. 

## 5. Implications 

Genomic services are inconsistently used to screen for LS within VHA clinical care; our findings highlight three areas important for practice change that could be targeted to improve use of genomic services in VHA for CRC, presented in Table 3. One area is expectation that identifying LS is a priority, targeted at a structural level through policy to standardize methods for collecting family history and testing tumors and to guide clinicians in counseling probands whose family members will need to access testing outside of the VHA system. Another area is targeting individual-level clinician knowledge about LS screening, which could be improved through education about evidence-based processes for identifying LS and ways to access services within the VHA. A third area involves enhancing genomic services themselves, specifically improving the availability of genomic expertise; for example, tools to facilitate guideline-informed use of FHH and a centralized genomic service, as the VHA has developed, can facilitate use of testing and counseling for LS, and this support for using genomic services is desired by clinicians. 

## Figures and Tables

**Table 1:** Semi-structured interview guide for key informants to evaluate awareness of and experience with genomic services for colorectal cancer care in the Veterans Health Administration.

Background	What is your current VHA position (manager, staff physician, service chief)? For how long have you been in your current position? For how long have you been at the VHA?
Availability	Please tell me what genomic services are available at your VHA facility to identify hereditary colorectal cancer. (ASK ABOUT: MSI/IHC analysis, genetic sequencing, and genetic counseling.)
Requesting Services	How are requests made? (*Probes*: Who makes the referral? Who approves the referral? How are the results documented? What are challenges? What is helpful?) IF PARTICIPANT MAKES REFERRALS FOR GENOMIC SERVICES, ASK: What factors help you decide which patients to refer [Specify what type of test: MSI, IHC, genetic sequencing]? (*Probes*: Age of diagnosis, presence of family history, known mutation in family.) Related to colorectal cancer, are there any tests you order reflexively, without request from gastroenterology or oncology?
Family History Documentation	How is family history documented in the medical record at your VHA facility? (*Probes*: When is it documented (e.g., initial visit to primary care, upon referral to specialist, at time of diagnosis)? Who documents it? Where do they document it? What information do they document? When family history is documented, how many generations are typically included?) What resources or systems are available for documenting family history (*Probe*: charts, forms, templates)? What reasons do you think clinicians would give for difficulty documenting family history? (*Probe:* for whether lack of standardization or specific place for family history documentation is a barrier, or whether the current system within the facility works despite lack of standardization.)
Making Referral Decisions	When a Veteran younger than age 50 is diagnosed with colorectal cancer, what services are considered standard at your facility as the next step in that patient’s care? If no mention of Bethesda guidelines or Amsterdam criteria, ask: Are you familiar with the Bethesda Guidelines and the Amsterdam Criteria? (NOTE: See criteria within this document; can review with respondent as needed)
Informing Patient	How are patients informed of results? (*Probes:* Who is responsible for discussing results with the patient? Which visit (primary care, oncology, gastroenterology)? What resources are available at your facility to support the clinician in interpreting results and informing the patient? What steps are taken to inform family members of the results?)
Informing Care	IF NON-VHA GENOMIC SERVICE, ASK: How are results reported back to the VHA facility? (*Probes*: How are the results documented? What time is required to receive the results? How are clinicians informed of the results?) How are the results of a genomic test (NOTE: specify if molecular test or genomic sequencing) used to inform a plan for preventive or prophylactic intervention for CRC?
	Do you have any comments regarding services related to hereditary colon cancer that you would like to add? **That completes the interview. Would you be able to suggest anyone else at your facility who might be knowledgeable about the evaluation of hereditary colorectal cancer? Thank you very much for your time.**

**Table 2:** Domains and constructs based on the Anderson Behavioral Model of Health Services ^1^ and the Diffusion of Innovation Model ^2^ used to analyze qualitative data on genomic services for colorectal cancer care in the Veterans Health Administration.

Domain	Construct	Definition
*Structural*	Availability ^1^	Whether genomic services are perceived as being available at facility, regardless of whether or not used in-house
	Innovation-system fit ^2^	Fit with the organization’s existing values, norms, goals, skill mix, ways of working; an aspect of system readiness for use of genomic services
	Incentives and mandates ^2^	Structural-level diagnostic and treatment guidelines, policies and procedures related to patient care
	Interorganizational networks ^2^	Linkages through common structures and explicit shared values and goals
*Individual*	Psychosocial factors (attitudes, knowledge) ^3^	Extent to which clinicians value incorporating genomics into colorectal care or demonstrate knowledge and interest; predisposing factor
	Enabling factors ^1,3^	Resources that support clinicians’ use of genomic services
*Innovational*	Relative advantage ^2^	Clear, unambiguous advantage in effectiveness of genomic services
	Augmentation/Support ^2^	Whether or not the genomic service comes with features to facilitate use, including templates, training, experts

^1^ Phillips (1998, [15]); ^2^ Greenhalgh (2004, [14]); ^3^ Bradley (2002, [13]); definitions adapted for this study.

**Table 3:** Overview of barriers and facilitators to adoption of genomic services for colorectal cancer care in the Veterans Health Administration.

Domain	Genomic Service			Implication
	*Family Health History (FHH) Documentation*	*Clinical Testing (Molecular and Genetic)*	*Genetic Counseling*	
Structural	+/− FHH routinely collected; however, lack of policy to standardize collection according to guidelines (incentives and mandates ^1^)	− Limited use and, for some, unclear referral process due to perceived low disease burden (innovation-system fit ^1^)	+/− GMS has facilitated referral process for genetic counseling; however, limited access presently (inter-organizational networks ^1^)	Structural barriers are lack of mandates for guideline adherent FHH documentation, perceived low need for testing in VHA patient population, and limited availability of in-house genetic counseling service; national VHA LS policy could improve adherence to guidelines via mandates for systematic FHH documentation, clinical testing protocol, and genetic counseling access.
Individual	+/− Gastroenterologists and oncologists likely to consider FHH documentation for CRC; however, when documented, completed with limited detail (attitudes ^2^)	+/− Across specialties, clinician use of testing facilitated by individual awareness/interest and local champion, and negatively impacted by lack thereof (attitudes ^2^; resources ^2,3^)	+ Resources facilitative because variability in individual expertise (attitudes ^2^; resources ^2,3^)	Individual-level barriers for FHH and clinical testing are low knowledge of or interest in LS, particularly by non-specialists; development of clinician education, local clinical champions, and genetic counseling resources could increase knowledge of evidence-based processes and ways to access services.
Innovational	− Lack of template/tool to facilitate guideline-informed use of FHH (augmentation/support ^1^)	+ Perceived advantage of molecular testing as more reliable 1st step than FHH (relative advantage ^1^)	+ “Expert” genomics support appreciated, such as provided by academic affiliate or within VA by local navigators, other VA facilities, or centralized GMS (augmentation/Support ^1^)	Availability of a tool (or template) could facilitate adherence to guidelines for documenting FHH. Clinical testing regarded as advantageous, indicating potential for wider uptake. Access to expert support is essential.

+ = facilitator; − = barrier; GMS = VHA Genomic Medicine Service; FHH = family health history; LS = Lynch Syndrome; EMR = electronic medical record; ^1^ Greenhalgh (2004, [14]); ^2^ Bradley (2002, [13]); ^3^ Phillips (1998, [15]).

## Abbreviations

The following abbreviations are used in this manuscript:

LS: Lynch syndrome
HNPCC: hereditary nonpolyposis colon cancer
CRC: colorectal cancer
MSI: microsatellite instability
IHC: immunohistochemistry
VHA: Veterans Health Administration
GMS: Genomic Medicine Service
